# Psychosis and enuresis during disulfiram therapy

**DOI:** 10.4103/0019-5545.31622

**Published:** 2006

**Authors:** P.A. Sherif, K. Krishna Murthy

**Affiliations:** * Resident, Department of Psychiatry, Fr. Muller Medical College, Mangalore 575002, Karnataka; ** Professor and Head, Department of Psychiatry, Fr. Muller Medical College, Mangalore 575002, Karnataka

**Keywords:** Disulfiram, psychosis, enuresis

## Abstract

Disulfiram is the drug that is commonly prescribed for the treatment of alcohol dependence syndrome, and transient functional psychosis has been reported as one of its side-effects. Enuresis is another rare adverse effect reported. This report discusses a case of acute psychosis and enuresis in a patient on disulfiram who had ingested alcohol.

## INTRODUCTION

In the treatment of alcohol dependence, it would seem highly unlikely that one and the same method of treatment should be the best for all. A comprehensive therapeutic intervention should be the correct approach as a number of interactive, predisponsing, precipitating factors have been at work to produce the condition. A brief, simple and noninvasive treatment in the form of disulfiram is being given in the management of alcoholism.

Disulfiram (tetra ethyl thiurium disulfide), a quaternary ammonium compound acts as a deterrent against drinking (if given 3–12 hours before an alcoholic drink). Like any other drug disulfiram also has side-effects. The commonly produced side-effects are hepatic, neurological, dermatological and psychological in nature. The infrequency of side effects with disulfiram confuses a person whether the side effects are caused by disulfiram or are by chance.

## THE CASE

Mr N., a 37-year-old unmarried man presented to the casualty department with complaints of smiling to himself, talking irrelevantly, being preoccupied and passing urine in his trousers.

He had been on disulfiram (250 mg hs) for the treatment of alcohol abuse for the past 8 months without his knowledge as he was not motivated to give up his drinking habit. Disulfiram had drastically reduced his alcohol consumption. Three days before presentation, he was administered 500 mg of disulfiram at night by his mother, who anticipated that he might consume alcohol the following day. The following morning he, along with his friends, went on a drinking binge of over 700 ml of alcohol, including whisky and rum. He was dropped home by his friends that night. The day after, he left for work and friends and relatives noticed changes in behaviour; talking irrelevantly at times, smiling to himself and being preoccupied. The same evening he drove off in a car for a test drive and did not return for 2 hours. This led to a search by his friends and brother and they found him in his car which was parked 3 km away from his workplace. He identified his friends but could not answer how and why he was there. He kept smiling in answer to questions put to him.

He was then driven to the casualty department by his friends. On the way to the hospital, he expressed a desire to pass urine and, getting out of the car, he removed his trousers and stood naked without passing urine. His friends made him wear his clothes again and this time he passed urine in his trousers.

At presentation, he identified the setting as a hospital. This was the only question he answered; he just smiled in answer to all other questions. He seemed preoccupied with his own thoughts and hence he was admitted and given inj. lorazepam 4 mg i.m. stat.

The following day, he revealed that he had had vivid hallucinations of a single female commanding him to do certain acts. He reported thinking that all his siblings and he were dead. He felt that such thoughts were put into his mind by some external agency. He also reported that his thoughts were being taken out of his mind but could not elaborate further.

### Investigations

The patient had no significant neurological and physical problems on examination. All baseline blood investigations were within normal limits. There was a slight rise in his SGOT/ SGPT levels (SGOT: 45, SGPT: 50 U/L).

There was no family history or past history of mental illness. Before this incident, he was well adjusted, with a tendency to make friends easily. He was kept in the hospital for 3 days, treated with benzodiazepines and supportive measures and then discharged. He was followed up for 8 weeks and no psychiatric illness was noticed.

## DISCUSSION

A transient psychosis which lasted for 3 days remitted when disulfiram was stopped and none of the symptoms recurred in 8 weeks. The follow-up is presented here.

For the past 35 years, disulfiram is the commonly prescribed drug for the treatment of alcoholism. It has been recognized for its potential, physical and psychological effects in alcoholic and non-alcoholic subjects.^1^ It produces sensitivity to alcohol, which results in a highly unpleasant reaction when the patient ingests even a small quantity of alcohol. This is known as disulfiram alcohol reaction and is due to the accumulation of acetaldehyde in the blood.

A transient functional psychosis, occasionally enuresis, and peripheral neuropathy have been reported as side-effects of disulfiram. Other reported side-effects are confusion, disorientation, loss of memory, agitation and social withdrawal.^2^ The biochemical mechanism of this drug reaction has been attributed to low levels of dopamine B hydroxylase (DBH), the inhibition of which has been experimentally shown to induce mood changes in normal volunteers.^3^ Male alcoholics who developed psychotic symptoms during disulfiram treatment^4^ were found to have low levels of amine and monoamine oxidase, suggesting DBH blockage. Disulfiram also blocks the synthesis and production of norepinepherine and dopamine, which might be related to the development of psychosis.^5^,^6^

In two studies, Krishna Murthy *et al.*^7^,^8^ reported psychosis in 5 out of 53 and 6 out of 52 patients on disulfiram.

## RECOMMENDATIONS

Patients should be informed of the type of reaction that will be encountered if alcohol is taken by itself or as a component of food or other products. It is suggested that every patient under treatment should carry an identification card stating that he or she is receiving disulfiram and describing the symptoms most likely to occur as a result of disulfiram reaction in case of alcohol is consumed while on treatment. In addition, this card should identify the attending physician or the institution to be contacted in case of an emergency.
